# miR-130a activates the VEGFR2/STAT3/HIF1α axis to potentiate the vasoregenerative capacity of endothelial colony-forming cells in hypoxia

**DOI:** 10.1016/j.omtn.2021.01.015

**Published:** 2021-01-20

**Authors:** Jasenka Guduric-Fuchs, Edoardo Pedrini, Judith Lechner, Sarah E.J. Chambers, Christina L. O’Neill, Joana Mendes Lopes de Melo, Varun Pathak, Rachel H. Church, Stuart McKeown, James Bojdo, Kiran J. Mcloughlin, Alan W. Stitt, Reinhold J. Medina

**Affiliations:** 1 Wellcome-Wolfson Institute for Experimental Medicine, School of Medicine, Dentistry, and Biomedical Sciences, Queen’s University Belfast, Belfast BT9 7BL, UK

**Keywords:** vascular repair, miRNA, hypoxia, angiogenesis, endothelial progenitor, ECFC, endothelial, endothelium, endothelial cell

## Abstract

Hypoxia modulates reparative angiogenesis, which is a tightly regulated pathophysiological process. MicroRNAs (miRNAs) are important regulators of gene expression in hypoxia and angiogenesis. However, we do not yet have a clear understanding of how hypoxia-induced miRNAs fine-tune vasoreparative processes. Here, we identify miR-130a as a mediator of the hypoxic response in human primary endothelial colony-forming cells (ECFCs), a well-characterized subtype of endothelial progenitors. Under hypoxic conditions of 1% O_2_, miR-130a gain-of-function enhances ECFC pro-angiogenic capacity *in vitro* and potentiates their vasoreparative properties *in vivo*. Mechanistically, miR-130a orchestrates upregulation of VEGFR2, activation of STAT3, and accumulation of HIF1α via translational inhibition of *Ddx6*. These findings unveil a new role for miR-130a in hypoxia, whereby it activates the VEGFR2/STAT3/HIF1α axis to enhance the vasoregenerative capacity of ECFCs.

## Graphical Abstract

**Figure:**
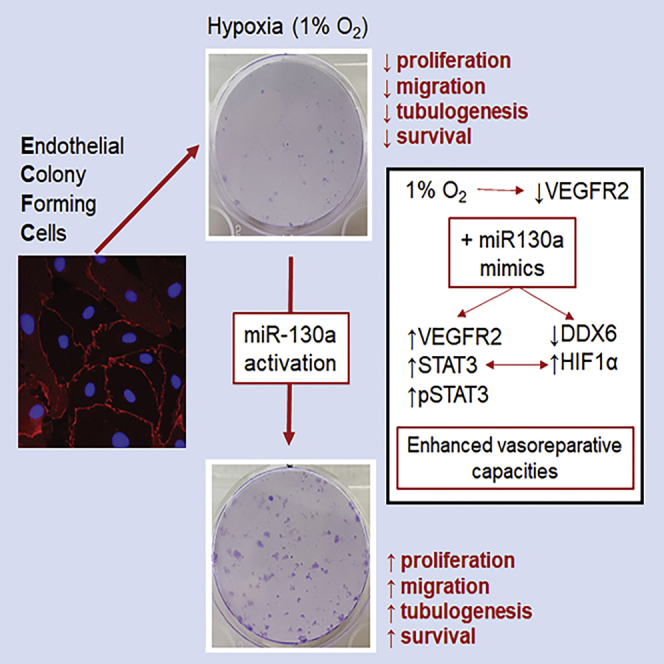

## Teaser

Cell therapies are often delivered into hypoxic tissues, which limits cell survival and engraftment. Guduric-Fuchs et al. demonstrate that miR-130a, enhanced endothelial colony-forming cells (ECFCs) function under hypoxia via activation of the VEGFR2/STAT3/HIF1α axis. miR-130a modulation in ECFCs offers an effective strategy to enhance their vasoreparative properties under hypoxia.

## Introduction

The ability of microRNAs (miRNAs) to repress translation of numerous target mRNAs makes them powerful regulators of cell physiology.^1^ A role for miRNAs in angiogenesis has been established^2^ where, for example, miR-23 and miR-27 drive pathological neovascularization.^3^ More recently, a miRNA network has been described for regulation of sprouting angiogenesis.^4^ Using microarray technology, a miRNA signature of hypoxia has been characterized^5^ and deep sequencing of endothelial cells has revealed a complex network of hypoxia-related miRNAs.^6^ Despite this progress, the modulatory role of miRNAs in endothelial cells exposed to hypoxia is unclear and such understanding would enable new strategies to regenerate the vasculature. With a key role in vascular homeostasis and regeneration, many endothelial progenitors have been described,^7^ albeit with some of these cell types not possessing endothelial differentiation capacity.^8^ Endothelial colony-forming cells (ECFCs) represent a subset of progenitors with an unequivocal endothelial phenotype and clonogenic potential^9^ and are consistently isolated from human umbilical cord blood (CB).^10^ Pre-clinical studies have demonstrated the capacity of ECFCs to repair damaged blood vessels in the ischemic heart,^11^ limb,^12^ and retina.^13^ In the context of cell therapy, ECFCs face hypoxic challenges when delivered into ischemic tissues and there is evidence to suggest that their functionality is impaired by hypoxia.^14^, ^15^, ^16^ New strategies to overcome the poor cell survival when delivered into ischemic tissue are needed to increase therapeutic efficiency of the next generation ECFC cell therapies.^17^

Cardiovascular diseases such as myocardial infarction, stroke, and peripheral artery disease are leading causes of death and morbidity worldwide.^18^ These diseases display progressive vascular insufficiency with ensuing oxygen deprivation and tissue ischemia. Cell therapy for tissue revascularization is emerging as a promising alternative to current treatments because restoring functional blood supply through vascular regeneration is critical for tissue repair. In the last two decades, there have been over 200 clinical trials for cell therapies in cardiovascular disease, and while most cell types are considered safe, therapeutic efficacy remains controversial.^19^ A major factor for such limited efficacy is poor cell retention and survival.^20^ Ischemic tissues display an imbalance in metabolic supply and demand, resulting in profound hypoxia.^21^ Cells delivered into ischemic tissues face a hypoxic microenvironment, which limits their performance, engraftment, and therapeutic potential.^22^ On the other hand, hypoxia has been reported to induce myocardial regeneration in zebrafish and adult mice;^23^^,^^24^ however, a meta-analysis of randomized controlled clinical trials evaluating remote ischemic preconditioning prior to cardiac surgery did not demonstrate clinical benefit.^25^ Since hypoxia preconditioning was shown to enhance the neovascularization capacity of human endothelial progenitors, including ECFCs,^26^^,^^27^ we hypothesized that transient manipulation of miRNAs involved in hypoxia may enhance pro-survival and pro-angiogenic pathways in ECFCs by simulating hypoxia preconditioning. Here, we show that activation of miR-130a provides ECFCs with an enhanced therapeutic efficacy in the setting of hypoxia via stimulation of the VEGFR2/STAT3/HIF1α axis. Transplantation of ECFCs primed *ex vivo* with mir-130a mimics improved their revascularization capacity.

## Results

### Hypoxia diminishes ECFC functionality required for vascular repair

**Figure 1:**
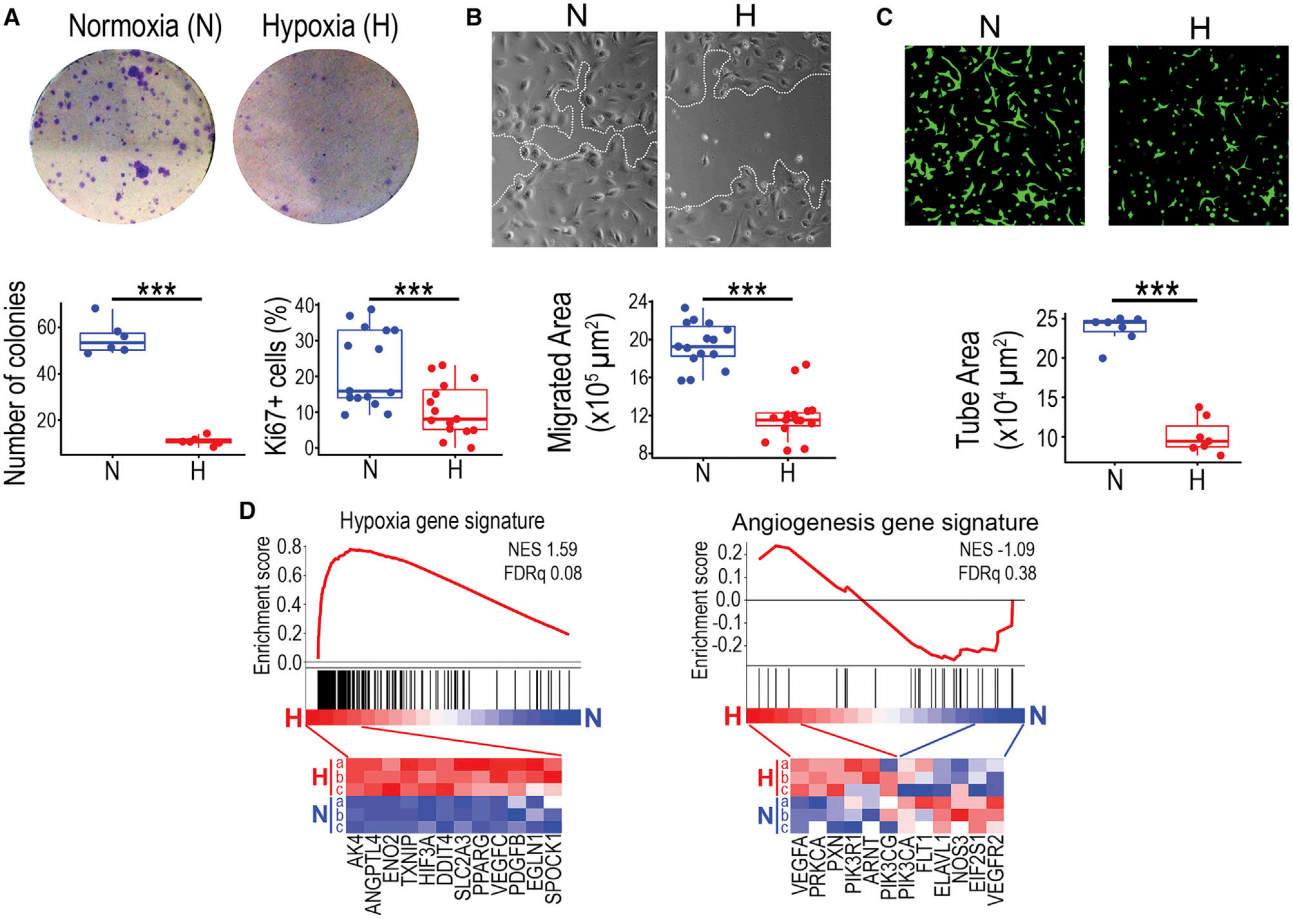
Effects of hypoxia on ECFC *in vitro* functionality and transcriptome changes (A) Clonogenic assays and Ki67 staining were performed on ECFCs under normoxic and hypoxic conditions. Crystal violet staining used for visualization and quantification of colony numbers. Quantification of Ki67-positive cells shown as percentage. (B) Micrographs for scratch wound migration assays. White dotted lines indicate ECFC monolayer migrating leading edge. Quantification of migrated area depicted as μm^2^. (C) Images of Matrigel 3D tube-formation assay. ECFCs stained in green with calcein. Quantification of tube area in μm^2^. (D) GSEA using ECFC transcriptome data comparing normoxia versus hypoxia and based on hypoxia and angiogenesis gene signatures. Heatmaps showing some of the transcripts significantly upregulated in ECFCs under hypoxia. Data in (A)–(C) presented as boxplots. ∗∗∗p < 0.001 (Student’s t test).

To investigate the effects of hypoxia in human ECFCs, we followed recent consensus guidelines for angiogenesis assays.^28^ Cells were exposed to hypoxia (1% O_2_) and then angiogenesis-related readouts including proliferation, migration, and 3D vascular morphogenesis were assessed. Under hypoxic conditions, ECFCs consistently declined in their cellular performance as demonstrated by a significant decrease in clonogenic potential and proliferative capacity (Figure 1A), 2D migration (Figure 1B), and 3D tube formation (Figure 1C). A hypoxic stimulus consistently reduced ECFC tube-forming capacity at 24, 48, and 72 h (Figures S1A and S1B). Next generation sequencing (NGS) transcriptome analysis performed after 24 h exposure to 1% O_2_ revealed a number of hypoxia-linked pathways and cell processes (Figure S1C). This was further confirmed by gene set enrichment analysis (GSEA) showing a clear enrichment of a hypoxia gene signature linked to endothelial cells (GSEA M259), with upregulation of genes such as *Angptl4, Eno2, Txnip, and Slc2a3* (Figure 1D). Interestingly, we did not find an enrichment of an angiogenesis gene signature (GSEA M12975), because while some genes such as *Vegfa, Prkca, and Pxn*were upregulated, others like *Vegfr2, Nos3, and Flt1* were downregulated (Figure 1D). Similar findings were obtained when using the Signaling Pathway Impact Analysis (SPIA) and the Ingenuity Pathway Analysis (IPA), which also highlighted a significant enrichment for the HIF1 pathway but not for the VEGF pathway (Tables S1 and S2). Therefore, GSEA, SPIA, and IPA results indicated an unequivocal gene enrichment for a hypoxic response, as expected, but not for angiogenesis.

**Figure 2:**
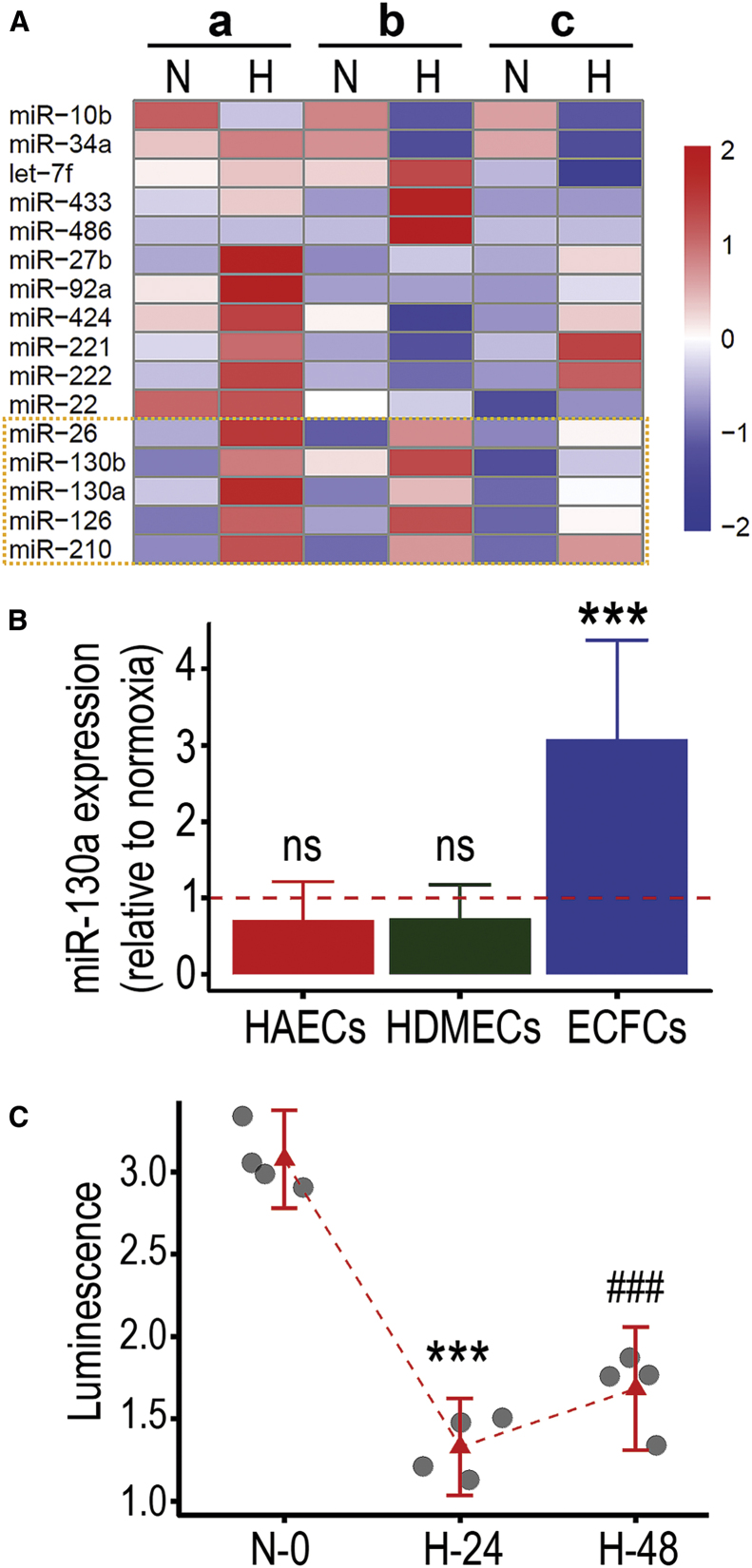
miR-130a expression and activity are increased in ECFCs exposed to hypoxia (A) qRT-PCR Taqman screening of miRNA profile changes in ECFCs after 24 h exposure to hypoxia. Biological replicates depicted as a, b, c, in normoxia (N) and hypoxia (H). Changes in miRNA expression level shown by color log scale as follows: red for upregulation, white for no change, and blue for downregulation. Dotted yellow box highlights miRNAs that are consistently upregulated across biological replicates. (B) Bar plots for miR-130a expression levels comparing normoxia and hypoxia conditions in human aortic endothelial cells, human dermal microvascular endothelial cells, and ECFCs. ∗∗∗p < 0.001 (ANOVA). (C) miR-130a 3′ UTR luminescence reporter system to evaluate miR-130a activity under normoxia and after 24 and 48 h of hypoxia. Dot plots with 95% confidence interval (CI). ∗∗∗p < 0.001; ###p < 0.001; ns, not significant (ANOVA).

We then performed a miRNA screening in ECFCs exposed to hypoxia using Taqman probes for 16 miRNAs previously reported in endothelial cells (Figure 2A). miR-10b was downregulated in hypoxia while miR-26, miR-130a, miR-130b, miR-126, and miR-210 were upregulated. Among these altered transcripts, miR-210 and miR-26 have already been reported to be hypoxia-inducible miRNAs (known as hypoximiRs).^29^^,^^30^ miR-126 has been studied as endothelial specific with roles in developmental angiogenesis.^31^ miR-130a is much less studied in relation to endothelial cell biology, although one report has suggested a link to angiogenesis;^32^ therefore, we decided to focus our attention on this miRNA. To elucidate whether miR-130a expression was upregulated in other mature endothelial cells exposed to hypoxia, we tested primary human endothelial cells from the aorta and skin and found that these did not upregulate miR-130a (Figure 2B). In addition, miR-130a was not found upregulated in HUVECs exposed to hypoxia based on an *in silico* analysis of GEO dataset GSE17944^33^ (Figure S2A). To confirm miR-130a bioactivity in ECFCs exposed to hypoxia, we utilized a synthetic miR-130a 3′ UTR luminescence reporter system. We found a significant decrease in luminescence signal in ECFCs transfected with this vector after 24 and 48 h of hypoxia exposure (Figure 2C), indicating increased miR-130a bioactivity in hypoxic conditions. Taken together, our results confirmed that hypoxia severely impairs ECFC functionality, which occurs concomitantly with upregulation of miR-130a expression and bioactivity.

### miR-130a overexpression enhances ECFC function under hypoxia

**Figure 3:**
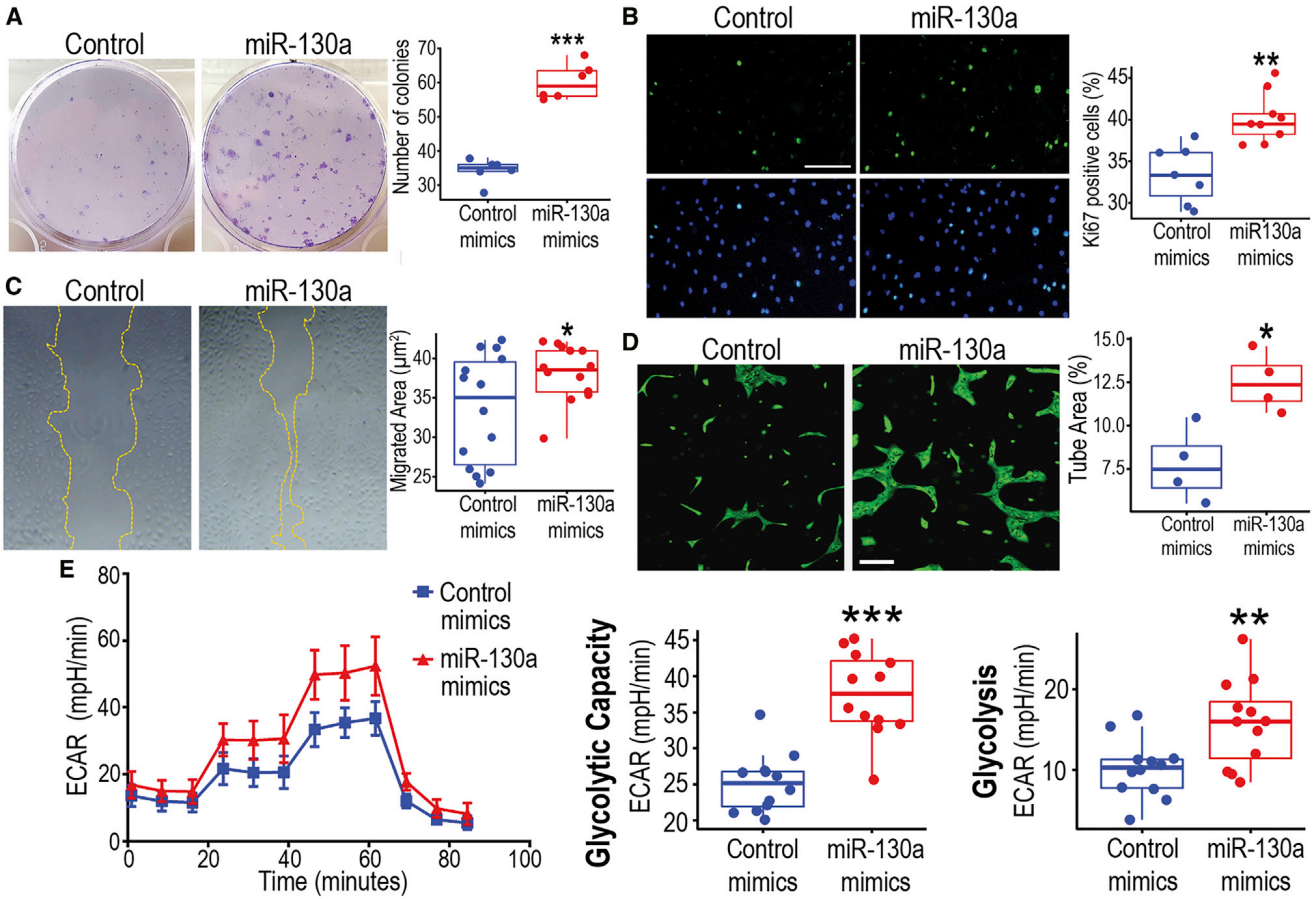
ECFCs treated with miR-130a mimics exhibit enhanced functionality (A) Clonogenic assay under hypoxia to compare ECFCs overexpressing miR-130a with controls. Crystal violet staining was used to visualize and quantify colony number. (B) Immunofluorescent staining for Ki67 in green, and nuclei stained in blue with DAPI. Scale bar, 300 μm. Quantification of Ki67-positive cells as percentage. (C) Micrographs for scratch wound migration assays under hypoxia. Yellow dotted lines indicate the migrating edge of ECFC monolayer. Quantification of migrated area depicted as μm^2^. (D) Images of Matrigel 3D tube-formation assay, ECFCs stained in green with calcein. Scale bar, 200 μm. Quantification of tube area as percentage of the total area. (E) Glycolysis stress test to evaluate glycolysis through changes in the extracellular acidification rate (ECAR) using the Seahorse XF analyzer with consecutive injections of 10 mM D-glucose, 1 μM oligomycin, and 50 mM 2-deoxyglucose at 20, 40, and 60 min, respectively. Boxplots generated with data from Seahorse XF Report Generator software. Data in (A)–(E) presented as boxplots. ∗∗∗p < 0.001; ∗∗p < 0.01; ∗p < 0.05 (Student’s t test).

To establish a mechanistic link between miR-130a and ECFC responses to hypoxia, we used miRNA mimics or locked nucleic acid (LNA)-miRNA inhibitors to modulate miR-130 activity in these cells. We asked whether overexpression or downregulation of miR-130 could modulate the ECFC response to hypoxia. ECFCs were transfected with miR-130a mimics or scrambled control mimics. miR-130a overexpression was confirmed by Taqman PCR (Figure S2B) and then *in vitro* functional assays were performed under 1% O_2_. Cell function assessment, comparing ECFCs transfected with miR-130a mimics versus scrambled control mimics, highlighted a remarkable improvement in the clonogenic potential (Figure 3A), proliferative capacity (Figure 3B), and survival (Figure S3A) with miR-130a mimics. In addition, miR-130a mimics-treated ECFCs exhibited enhanced 2D migration capacity (Figure 3C), with a significant increase in wound closure rate and faster ECFC migratory speed (Video S1). In agreement with these findings, there was also a significant increase in 3D tube formation observed in ECFCs overexpressing miR-130a under hypoxia (Figure 3D). In contrast, when we used LNA miR-130 inhibitors, this further exacerbated the decrease in ECFC proliferative and migratory capacity induced by hypoxia (Figures S3B and S3C), which suggests that the miR-130a increase is a compensatory protective mechanism in hypoxic ECFCs. Importantly, under normoxia, miR-130a overexpression using mimics did not enhance ECFCs proliferative, migratory, and tube-forming capacities (Figure S4), a response that underscores an important role for miR-130a solely in hypoxic conditions. Since angiogenic responses in endothelial cells are known to be highly dependent on shifts toward glycolytic metabolism,^34^ we evaluated cellular bioenergetic changes in ECFCs exposed to hypoxia. There was a significant increase in basal glycolysis and glycolytic capacity in ECFCs transfected with miR-130a mimics when compared to scrambled mimics (Figure 3E). Overall, these results from cellular functional assays *in vitro* confirmed that miR-130a activation in ECFCs enhanced their angiogenic performance when exposed to hypoxic conditions.

Video S1. miR130a mimics treatment increased ECFC migration speed under hypoxia Dynamics of cell migration studied in ECFCs under hypoxia using the IBIDI silicone culture inserts. ECFCs were stained with CytoPainter Cell Tracking Green and snapshots were taken every 15 min for 12 h in EVOS onstage incubator.

### Enhancement of ECFC functionality induced by miR-130a in hypoxia is driven by the VEGFR2/STAT3/HIF1α axis

**Figure 4:**
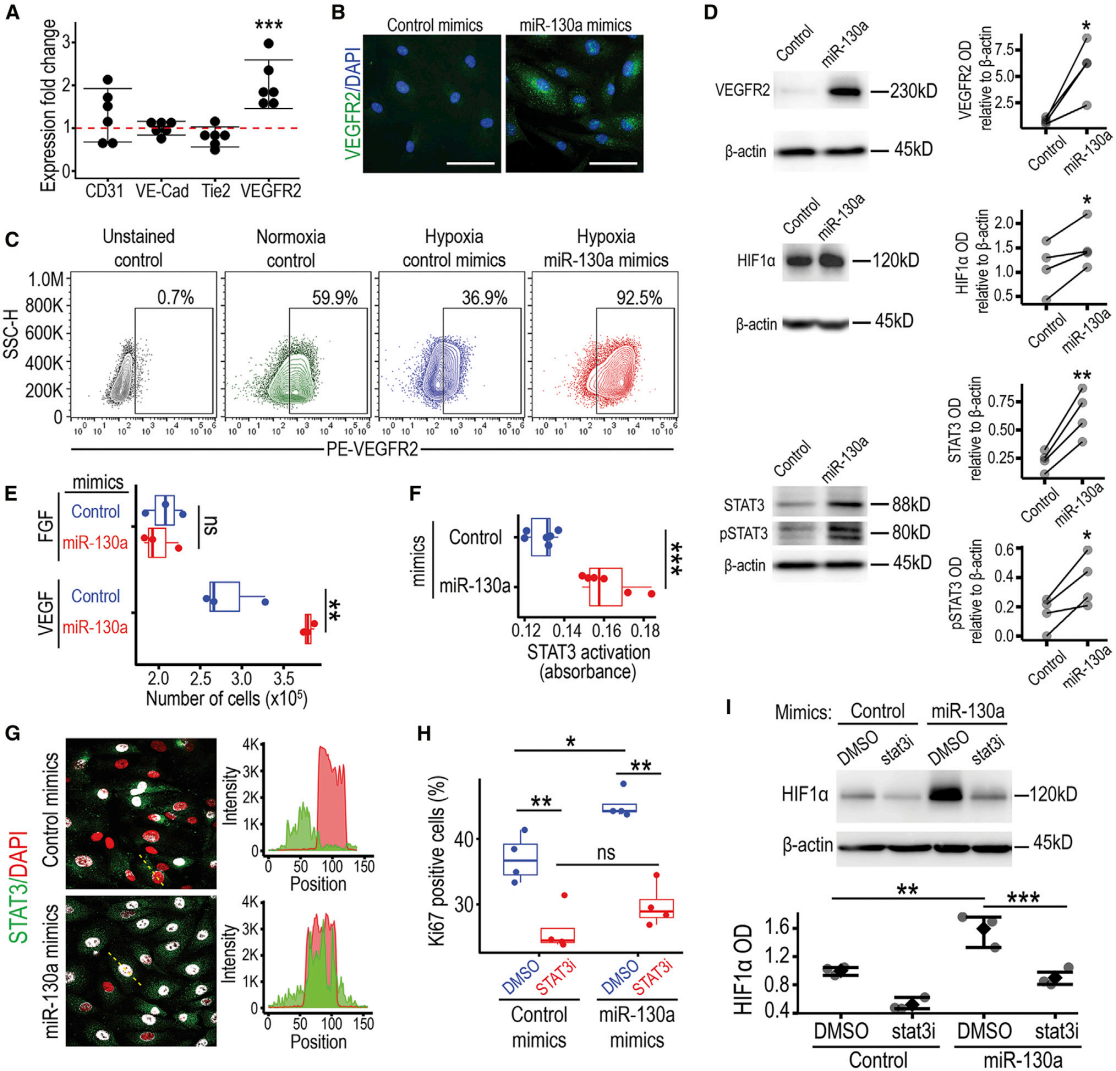
miR-130a activates the VEGFR2/STAT3/HIF1 signaling axis in ECFCs under hypoxia (A) qRT-PCR assessment of angiogenesis-related genes in ECFCs from six biological replicates. ECFCs transfected with miR-130a mimics or control mimics were exposed to hypoxia for 48 h prior to RNA extraction, and their gene expression was compared. Data are presented as dot plots with 95% CI, ∗∗∗p < 0.001 (ANOVA). (B) Immunofluorescent staining for VEGFR2 in green, and nuclei are stained in blue with DAPI. Scale bar, 100 μm. (C) Flow cytometry analysis of VEGFR2 cell surface expression. Gates highlight the positivity threshold established using unstained controls. Percentage of positivity shown on the top right corner. (D) Western blot analysis of VEGFR2, HIF1α, STAT3, and phosphorylated STAT3 in the protein extract of miR-130a and control mimic transfected ECFCs exposed to hypoxia. ImageJ-based densitometry was used for quantification and statistical analysis. ∗∗p < 0.01; ∗p < 0.05 (Paired t test). See also Figure S6. (E) Graph showing cell counts after plating 1 × 10^5^ of ECFCs and culturing for 3 days in hypoxia in basal media supplemented with either FGF or VEGF. Data are presented as bar plots. ∗∗p < 0.01; ns, not significant (Student’s t test). (F) Levels of nuclear STAT3 bound to its DNA target oligo sequence measured by ELISA-based TransAM STAT3 kit as absorbance. Data are presented as boxplots, ∗∗∗p < 0.001 (Student’s t test). (G) Immunofluorescent staining for STAT3 in green and nuclei stained with DAPI in red to highlight increased nuclear localization in the miR-130a mimic-transfected ECFCs. Colocalization shown in white. (H) Quantification of Ki67-positive cells as percentage of the total cell population. ECFCs transfected with miR-130a mimics or controls were treated with STAT3 inhibitor S31-201(stat3i). Data shown as boxplots. ∗∗p < 0.01; ∗p < 0.05 (ANOVA). (I) Western blot analysis of HIF1α in miR-130a mimics-treated ECFCs cultured with stat3 inhibitor. ***p < 0.001; **p < 0.01 (ANOVA).

To understand the molecular mechanism by which miR-130a enhances ECFC functionality under hypoxia, we evaluated expression of genes related to endothelial function by quantitative reverse-transcriptase polymerase chain reaction (qRT-PCR). After miR-130a overexpression in hypoxia, we found upregulation of *Vegfr2* transcript, but no change in CD31 (*Pecam1*), *VE-cadherin*, and *Tie2* (Figure 4A). Similarly, an increase in VEGFR2 protein expression was shown by immunocytochemistry following miR-130a mimic transfection (Figure 4B). These data were further corroborated by flow cytometry analysis that highlighted a decrease in VEGFR2 expression from 60% in normoxia to 37% under hypoxia and a robust VEGFR2 increase to 93% after miR-130a overexpression in hypoxia (Figure 4C). There were no changes in the expression levels of CD31, CD105, CD146, CD157, and CD201; however, miR-130a overexpression in hypoxia consistently upregulated cell surface expression of CD34 (Figure S5). The miR-130a induction of VEGFR2 protein expression was significantly increased in protein lysates (Figure 4D). In addition, miR-130a mimics also significantly increased the expression of HIF1α (Figure 4D; Figure S6). Since STAT3 activation is an essential downstream component for VEGFR2 signal transduction pathway in endothelial migration and tube formation,^35^ we performed western blot analyses after miR-130a mimic transfection to evaluate total and phosphorylated STAT3. We found that increased protein levels of VEGFR2 were accompanied with significant increases in both total and phosphorylated STAT3 (Figure 4D; Figure S6). The VEGFR2 increase induced by miR-130a had functional implications since ECFCs transfected with miR-130a mimics showed an enhanced proliferative response to VEGF but not to fibroblast growth factor (FGF) in hypoxia, when compared to ECFCs transfected with scrambled control mimics (Figure 4E). To further explore the biological relevance of STAT3 pathway in relation to miR-130, we evaluated STAT3 activation by the TransAM DNA binding assay, which showed higher STAT3 binding to its target DNA sequence in miR-130a transfected ECFCs under hypoxia (Figure 4F). These results were in line with STAT3 subcellular localization by immunofluorescence, which confirmed increased STAT3 nuclear translocation in miR-130a overexpressing ECFCs under hypoxia (Figure 4G). To evaluate whether this STAT3 increase and activation was essential for miR-130a enhancement of ECFC proliferation under hypoxia, we used the STAT3 inhibitor S31-201 (STAT3i). When miR-130a overexpressing ECFCs were treated with 30 μM STAT3i, the effect of miR-130a on ECFC proliferation under hypoxia was abolished (Figure 4H). Interestingly, STAT3 is known to inhibit HIF1α degradation^36^ and activate HIF1 target genes.^37^ Therefore, we evaluated this and found that in hypoxic ECFCs, STAT3 inhibition significantly reduced miR-130a mimics enhanced HIF1α expression (Figure 4I). This suggests that STAT3 may play a role in HIF1α stabilization. In agreement with these results, qRT-PCR showed that ECFCs transfected with miR-130a mimics exhibited a higher expression of HIF1 downstream targets *Epo* and *Glut1* (Figure S7). These results indicate that the enhancement of ECFC functionality by miR-130a under hypoxia is mediated by the activation of VEGFR2, STAT3, and HIF pathways.

### miR-130a targets DDX6 in hypoxic ECFCs

**Figure 5:**
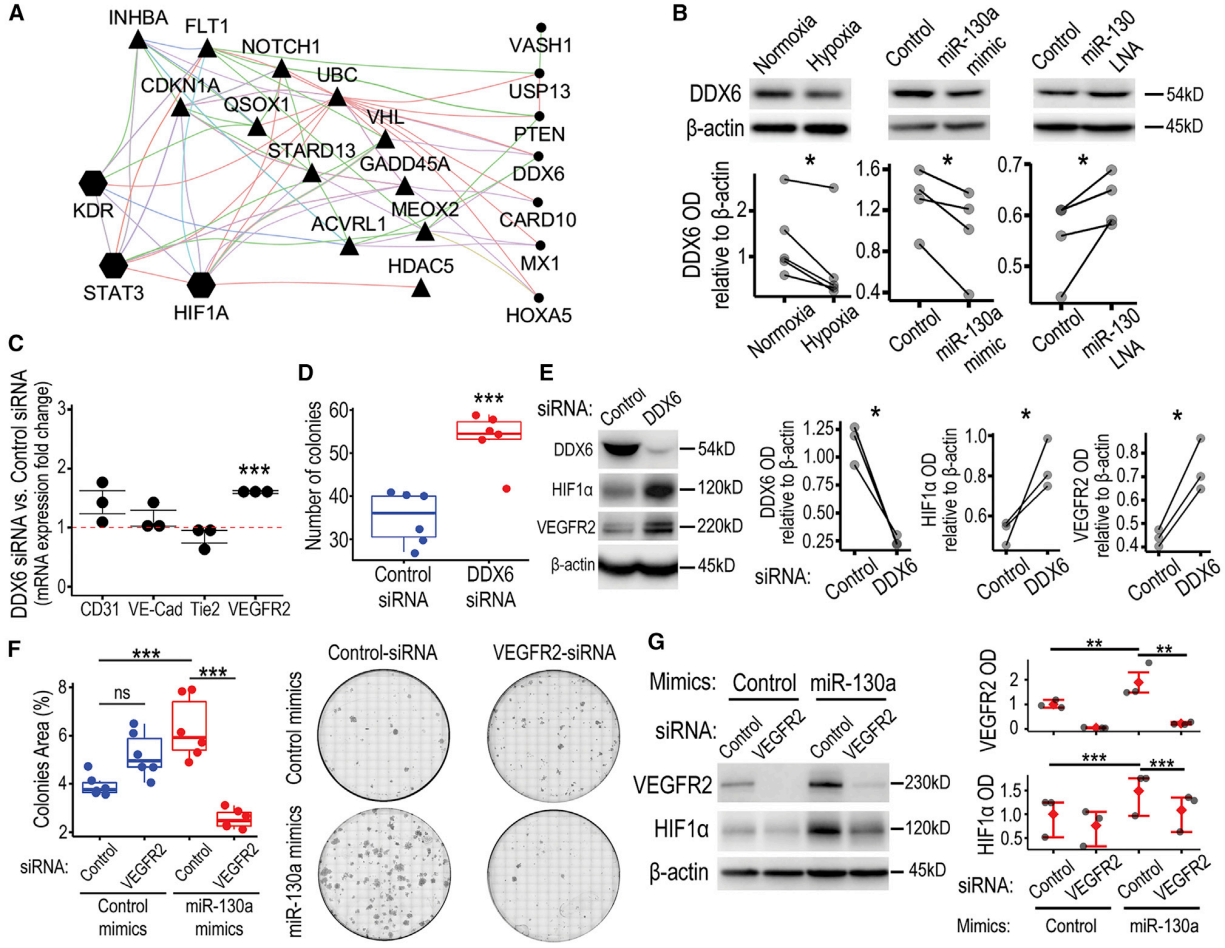
Inhibition of the miR-130a target DDX6 in hypoxia phenocopies the effects of miR-130a overexpression (A) Interactome generated by Cytoscape-Genemania between miRNA-130a target genes associated with negative regulation of angiogenesis and the VEGFR2/STAT3/HIF1α axis. Triangles highlight miR-130a targets with a direct association with the pathway while circles highlight the ones that need an intermediary node. Edge color indicates type of association as follows: purple for co-expression, blue for colocalization, green for genetic interactions, light blue for pathway, pink for physical interactions, orange for predicted interaction, and brown for shared protein domains. (B) Western blot for DDX6 protein expression in ECFCs under hypoxia, treated with miR-130a mimic or miR-130 LNA. ImageJ-based densitometry of bands is depicted in relation to β-actin. ∗p < 0.05 (Paired t test). See also Figure S9. (C) qRT-PCR assessment of gene expression, comparing DDX6-siRNA transfected ECFCs with control-siRNA transfected cells, after 48 h in hypoxia. Data are shown as dot plots with standard error of the mean. ∗∗∗p < 0.001 (ANOVA). (D) Quantification of number of colonies in the clonogenic assay under hypoxic conditions comparing DDX6-siRNA transfected ECFCs with control-siRNA transfected cells. Data are shown as boxplots. ∗∗∗p < 0.001 (Student’s t test). (E) Western blot analysis of HIF1α and VEGFR2 expression in ECFCs with silenced DDX6. ∗p < 0.05 (Paired t test). See also Figure S10. (F) Quantification of number of colonies in the clonogenic assay under hypoxic conditions comparing VEGFR2-siRNA transfected ECFCs with control-siRNA transfected cells. Data shown as boxplots. ∗∗∗p < 0.001 (Student’s t test). Representative images are shown. (G) Western blot analysis of HIF1α expression in ECFCs with silenced VEGFR2. ***p < 0.001; **p < 0.01 (ANOVA). See also Figure S11.

To explore the possible regulatory role of miR-130 on the VEGFR2/STAT3/HIF1α axis, we sought to identify the most relevant binding targets of this miRNA. First, we used the mirWalk database to identify predicted miR-130a-3p targets and the Molecular Signature Database (MSigDB) from the Broad Institute to identify angiogenesis-related gene sets. Among the 5,316 retrieved genes from mirWalk and the 612 genes from MSigDB, we focused on 161 common ones that were miR-130a targets and angiogenesis-related. We further selected 19 genes that negatively regulate angiogenesis and may explain miR-130a enhancement of endothelial function. An interactome network was generated by inputting these 19 genes and VEGFR2/STAT3/HIF1α into Cytoscape with the Genemania plugin (Figure 5A). This highlighted distinct direct and indirect associations between miR-130a target genes and VEGFR2/STAT3/HIF1α. Triangles depict nodes with direct associations, such as FLT1 and UBC, which relate to the three proteins in the pathway of interest, while others like ACVRL1 and QSOX1 are VEGFR2 (KDR)-specific. We also identified nodes with indirect associations in circles, such as DDX6, PTEN, and CARD10. Additionally, we took only the validated miR-130a targets, which were 185 from the 5,316 identified, to perform an unbiased process enrichment analysis. This identified an enrichment of genes involved in regulation of cytoskeleton organization, protein stability, and protein deubiquitination. The top 9 clusters were rendered as a network (Figure S8), and interestingly included DDX6 (triangle) that was also identified in the angiogenesis-biased analysis. DDX6 is an RNA helicase with roles in cellular stress and hypoxia,^38^ processing (P)-body homeostasis regulating stem cell plasticity,^39^ and it is also targeted by miR-130a, thereby regulating HIF1α translation.^40^ Based on data from *in silico* analysis and evidence from published literature, we decided to determine whether DDX6 acted as a direct target for miR-130a in hypoxic ECFCs. Target prediction software seedVicious v1.3 corroborated results by identifying canonical target sites through the miR-130a seed region and five distinct marginal binding sites with estimated free energies for the annealing ranging from −8 to −3.89 kcal/mol. Next, we investigated the kinetics of DDX6 protein expression in ECFCs under hypoxia and found that DDX6 was downregulated in hypoxia at 24 h (Figure 5B; Figure S9), which matches the time when miR-130a bioactivity increased in hypoxia (Figure 2C). In addition, we characterized the role of miR-130a in modulating DDX6 expression in ECFCs under hypoxia, by overexpression or downregulation using miRNA mimics and LNA inhibitors, respectively. As expected, miR-130a overexpression led to a significant decrease in DDX6 protein expression (Figure 5B; Figure S9), phenocopying hypoxia-induced DDX6 decrease. On the contrary, miR-130 inhibition using LNA technology led to a significant increase in DDX6 protein levels (Figure 5B; Figure S9). Since miR-130a effectively decreased DDX6 protein expression, we investigated whether knockdown of DDX6 using small interfering RNA (siRNA) had similar effects to miR-130 overexpression. We found that silencing DDX6 in ECFCs exposed to hypoxia, led to upregulated *Vegfr2* transcript (Figure 5C) and a significant increase in clonogenic capacity (Figure 5D). DDX6 silencing significantly increased the expression of HIF1α and VEGFR2 (Figure 5E; Figure S10). This was in agreement with findings described for miR-130a overexpression and suggests that DDX6 plays a mechanistic role on miR-130a effects observed in ECFCs under hypoxia, which are associated with increased VEGFR2, HIF1α, and enhanced cell function. To further examine the importance of VEGFR2 in miR-130 effects, siRNA was used to silence VEGFR2 expression. Silencing of VEGFR2 completely abolished miR-130a induction of clonogenicity under hypoxia (Figure 5F) and significantly inhibited HIF1α expression (Figure 5G; Figure S11 ).

### miR-130a activation potentiates ECFC revascularization efficiency

**Figure 6:**
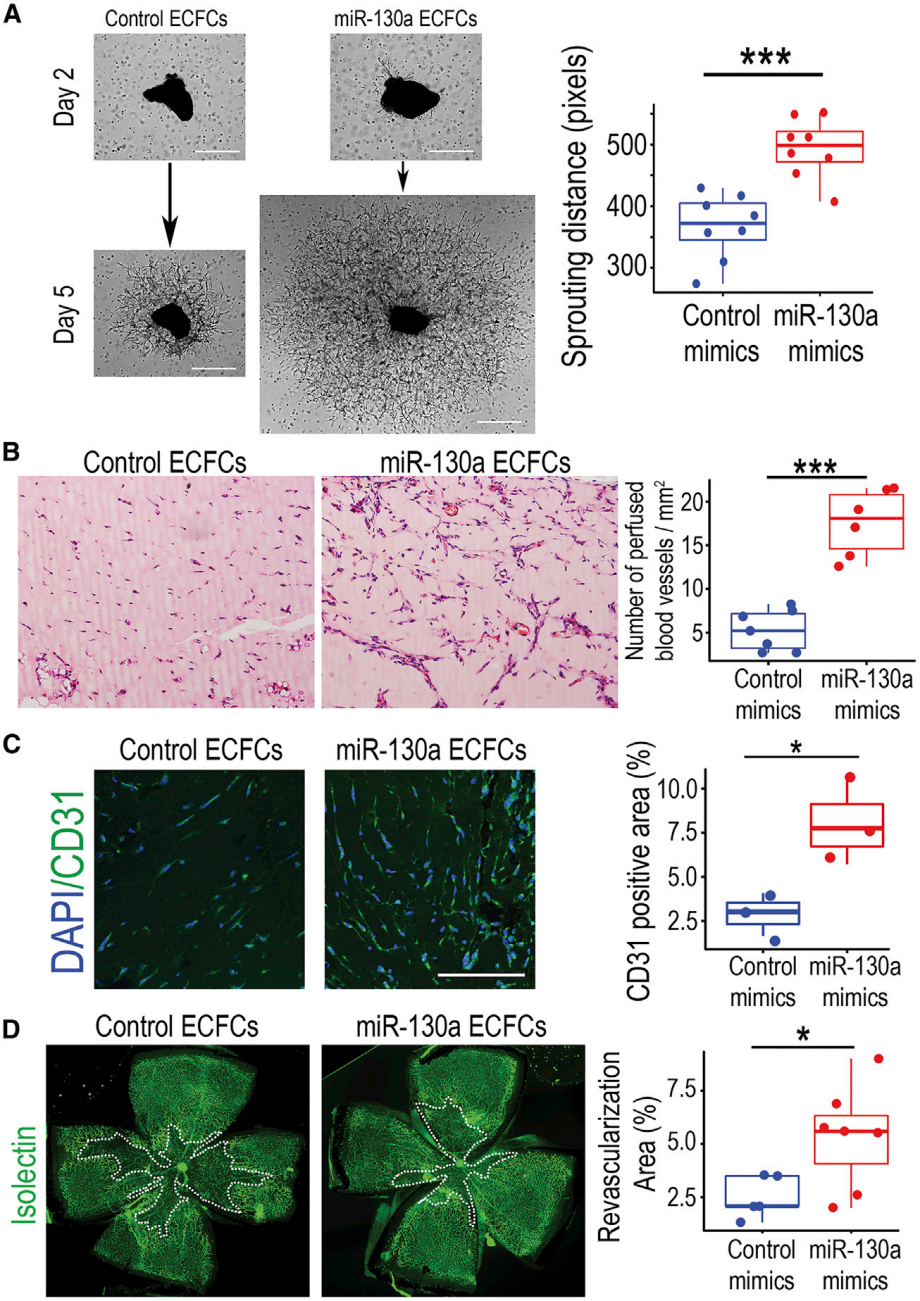
miR-130a enhances ECFCs angiogenic and vasoreparative function in *ex vivo* and *in vivo* models (A) Micrographs of *ex vivo* choroidal explants co-cultured with control or miR-130a overexpressing ECFCs quantified for sprouting angiogenesis at day 5. Scale bar, 500 μm. (B) Representative images of Matrigel implant sections stained with H&E. ECFCs transfected with miR-130a mimics or scrambled mimic controls were mixed with Matrigel and injected subcutaneously into nude mice. Matrigel plugs were retrieved 10 days after subcutaneous implantation for H&E staining. Quantification of the number of perfused vessels per mm^2^. (C) Immunohistochemistry of Matrigel implants with an antibody against CD31 to identify vasculature. (D) Representative images of flat-mounted retinas from the OIR model where ECFCs transfected with miR-130a or control mimics were delivered intravitreally at postnatal day 12 (P12; at the outset of central retinal ischemia). Retinas were sampled at P14, stained with isolectin to identify the retinal vasculature, and imaged for quantification of ischemic areas in the central retina. Data in (A)–(D) are presented as boxplots. ∗∗∗p < 0.001; ∗p < 0.05 (Student’s t test).

To determine whether miR-130a effects on ECFCs are applicable to more complex disease models, we used *ex vivo* and *in vivo* angiogenesis models. First, we examined ECFCs in the *ex vivo* choroidal explant model, where 1 mm diameter explants of murine choroid are suspended in Matrigel containing human ECFCs and the sprouting vessels are measured. Overexpression of miR-130a in ECFCs significantly increased sprouting distance (p < 0.001; Figure 6A). Second, we performed the subcutaneous Matrigel implant angiogenesis model in nude mice. Similar to the *in vitro* Matrigel tubulogenesis model (Figure 3D), ECFCs transfected with miR-130a mimics showed a significantly higher number of perfused vessels when compared to scrambled mimics-transfected ECFCs (p < 0.001) at 10 days after subcutaneous implantation (Figure 6B). In agreement with these, immunohistochemistry for CD31 using human-specific antibodies, within the Matrigel implants, demonstrated a higher vascular density for miR-130a overexpressing ECFCs than control mimics-ECFCs (Figure 6C). Third, we used a disease relevant *in vivo* model, the oxygen-induced retinopathy (OIR) model. We overexpressed miR-130a in ECFCs prior to delivery into the ischemic retina. The avascular area in the central retina was significantly decreased, when comparing ECFCs pre-treated with miR-130a mimics to scrambled control mimics (p < 0.05; Figure 6D). Taken together, this evidence suggests that activation of miR-130a in ECFCs is an effective strategy to enhance their cellular performance for vascular repair.

## Discussion

**Figure 7:**
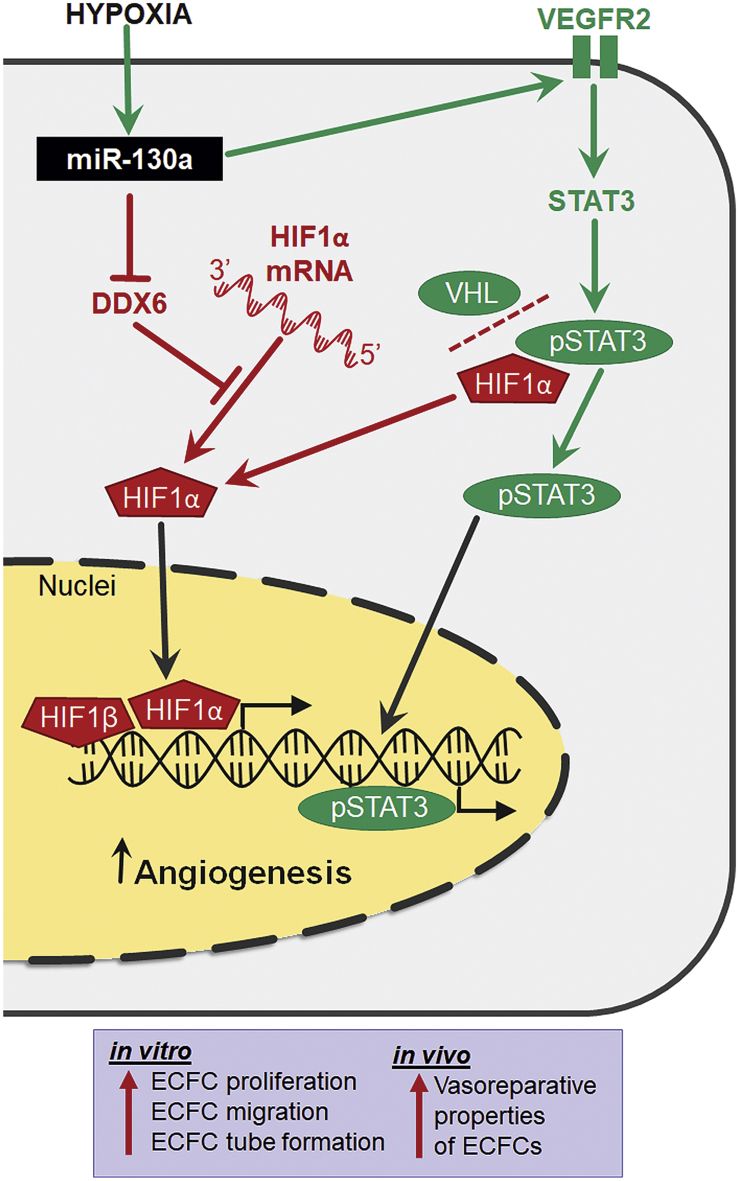
miR-130a regulates pro-angiogenic signals in ECFCs Schematic representation of the working model for miR-130a activation and function, under hypoxia, by modulating the VEGFR2/STAT3 and the DDX6/HIF1α molecular pathways to enhance ECFCs vasoreparative properties.

The miR-130/301 family has been recognized as a master miRNA regulator of pulmonary hypertension, a disease driven by hypoxia, and miR-130 upregulation was confirmed in endothelial cells isolated from murine hypoxic lungs.^41^ It was also reported that ECFCs can reside in pulmonary arteries and when isolated from patients with pulmonary artery hypertension (PAH), these cells exhibited higher proliferative capacity compared to non-PAH controls.^42^ Furthermore, the existence of ECFCs in PAH provides reinforcement of our findings, which demonstrate the proliferation-promoting effect of hypoxia in these cells through the miR-130 and VEGF pathway. It seems likely that this pathway plays a key role in the formation of plexiform vascular lesions in PAH. Importantly, this has potential for clinical translation, because intrajugular administration of human CB-derived ECFCs, in a rat model of bronchopulmonary dysplasia, was shown to restore lung function and attenuate pulmonary hypertension.^43^ Our findings revealed that miR-130a gain-of-function in endothelial progenitor ECFCs significantly enhanced their angiogenic potential under hypoxic/ischemic conditions *in vitro*, *ex vivo*, and *in vivo*. Here, based on multi-faceted evidence, we propose a novel regulatory pathway whereby miR-130a activates vasoreparative responses in ECFCs, through the VEGFR2/STAT3/HIF1α signaling axis (Figure 7). The current study has demonstrated that miR-130a increases VEGFR2 expression in the cell membrane of ECFCs. As the primary receptor that promotes angiogenesis-related signaling in endothelial cells, VEGFR2 is, at least partially, controlled by miR-130a and this serves to render ECFCs more responsive to VEGF ligand binding. Enhanced VEGFR2 signaling was also evidenced by the increase in STAT3 nuclear translocation and transcription factor activation. STAT3 appears to be a key downstream pro-angiogenic mechanism because its inhibition reduced endothelial proliferation beyond control levels.

A previous study suggested that miR-130a directly targets DDX6,^40^ and our results showed that the ECFC phenotype in DDX6 knockdown ECFCs is similar to miR-130a overexpressing cells. DDX6 is a DEAD box helicase whose function is critical for P-body assembly containing translationally repressed mRNAs.^39^^,^^44^ Evidence demonstrates that DDX6 acts as a molecular hub for translational repressors and mRNA decay enzymes within P-bodies.^45^ By diminishing DDX6 translation, miR-130a enhances HIF1α translation and therefore the HIF1-driven hypoxic transcriptional signature, which includes multiple pro-angiogenic genes such as *Vegfa*.^46^ Our study therefore identified a crosstalk between VEGFR2/STAT3 and DDX6/HIF1α as some of the downstream mechanistic effectors of miR-130a during hypoxia in ECFCs. This was demonstrated by VEGFR2 silencing modulating HIF1α expression, and conversely, DDX6 silencing modulating VEGFR2 expression. However, we cannot exclude the possibility of involvement of other pathways; for example, the miR-130/PPARγ/Apelin, which was described in hypoxic human pulmonary artery endothelial cells (HPAECs).^41^ Furthermore, our interactome analysis of miRNA-130a targets and angiogenesis identified other molecular pathways that may also play a role, such as ACVRL1 (Alk-1), FLT1 (VEGFR1), and CDKN1A (p21). While HPAECs upregulate miR-130a similarly to ECFCs under hypoxia, other human endothelial cells such as HUVECs, HAECs, and HDMECs do not show such responses. This serves to underline the importance of the role of miR-130a in some endothelial cells exposed to hypoxia but this is not ubiquitous in endothelium, especially since we know that there is considerable endothelial heterogeneity and specialization between vascular beds. Our findings recognized a robust upregulation of miR-130a in hypoxic ECFCs and therefore suggest the possibility that in ECFCs, miR-130a acts as a hypoximiR.

Hypoxia promotes angiogenesis in tumors and neovascular eye disease.^47^ However, it is important to highlight that this hypoxia-induced angiogenesis is largely VEGF-dependent.^46^ Our present work and other studies^14^, ^15^, ^16^^,^^48^ agree that constant exposure to 1% O_2_ diminishes ECFC function. Although hypoxia induced a modest *Vegfa* upregulation in ECFCs, there was a significant decrease in VEGFR2, explaining the loss of angiogenic function. We showed evidence that miR-130a activation increases VEGFR2 expression and restores a functional VEGF/VEGFR2 axis under hypoxia in the presence of VEGF.

A limiting factor for the success of cell therapeutics is the ischemic microenvironment, where injected cells are expected to survive, engraft, and perform, despite being deprived of oxygen and nutrients.^19^ *In vivo* ischemia significantly decreased ECFC angiogenic potential.^49^ Preconditioning of stem/progenitor cells is of particular importance in cardiovascular disease.^50^ Exposure of ECFCs to 2% O_2_ for 24 h was shown to accelerate their vascular repair properties.^27^ We propose that treatment of ECFCs with miR-130a mimics before transplantation is an alternative strategy to hypoxia preconditioning. Our evidence demonstrates that miR-130 manipulation in ECFCs effectively increases their performance under severe hypoxia (1% O_2_), and enables ECFCs to overcome microenvironmental challenges, such as occurs when the cells are delivered into diseased, ischemic tissues. It will be interesting to determine whether our miR-130a strategy is complementary to other reported methodologies to improve ECFC therapeutic potential. These include ECFC treatment with epigenetic drug Trichostatin A^51^ and priming with erythropoietin,^52^ erythropoietin mimetic,^53^ or soluble CD146.^54^^,^^55^ As a potential cell therapy for vascular regeneration, ECFCs have two mechanisms of action to enhance vascular repair; through direct cell incorporation^13^ into damaged vasculature and/or through release of paracrine pro-angiogenic soluble factors.^56^ Our findings demonstrate that miR-130a plays an important role in the hypoxia response in ECFCs, and its modulation can be used as a strategy to enhance the vasoreparative function of these cells when delivered into ischemic tissues as a therapeutic.

## Materials and methods

### Cell isolation and culture

ECFCs were isolated from umbilical CB of full-term pregnancies as previously described.^10^ The study has ethical approval by the NRES Committee with REC reference 15/YH/0281. CB mononuclear cell fraction was obtained by density gradient centrifugation and cells were plated on collagen-coated plates in EGM-2 medium (Lonza) supplemented with 10% fetal bovine serum (FBS). MNCs were cultured for up to 4 weeks with media changes every 24 h for the first week and 48 h thereafter. ECFC colonies usually appeared after 2–3 weeks of culture. ECFC identity was confirmed by flow cytometry. For hypoxia experiments, ECFCs at early passages (postnatal day 6 [P6] to P14) were cultured in a hypoxia chamber at 1% O_2_ and compared to normoxia (21% O_2_).

### RNA sequencing (RNA-seq), analysis, and data availability

Total RNA was isolated from cells using the Maxwell RSC simplyRNA Cells Kit (Promega) and a Maxwell RSC instrument (Promega) following the manufacturer’s instructions. Libraries were prepared using KAPA RNA HyperPrep Kit with RiboErase HMR (Roche). Samples were sequenced on a NextSeq500 instrument (Illumina) to yield at least 20 M reads per sample 75SE. Data were normalized and differential expression of genes identified using DESeq2.^57^ GSEA was performed using the fgsea package in R as an algorithm for preranked GSEA using cumulative statistic calculation. Data deposited to the NCBI Gene Expression Omnibus (GEO) database under GEO: GSE142123.

### Real-time qRT-PCR

Total RNA (including small RNAs) was isolated using the miRNeasy mini kit according to the manufacturer’s protocol (QIAGEN). RNA quantity and quality were assessed using the Qubit (Thermo Fisher Scientific) and Bioanalyzer (Agilent Technologies), respectively. RNA purity was assessed by NanoDrop. miRNA expression was assessed using TaqMan MicroRNA Assays (Thermo Fisher Scientific) according to the manufacturer’s protocol. For gene-expression analysis, complementary DNA (cDNA) was synthesized from 1 μg of RNA using a High-Capacity RNA-to-cDNA Kit (Thermo Fisher Scientific). The real-time PCR was performed using the Maxima SYBR Green qPCR mastermix (Thermo Fisher Scientific) in 10 μL reactions containing 2 μL of 1:10 cDNA dilution and 0.5 μM of gene-specific primers for 45 cycles in a LightCycler 480 (Roche).

### Cell transfection

Transfections were performed at 70% confluency using Lipofectamine RNAiMAX in EGM-2 media without antibiotic. MiRIDIAN miRNA mimics (Horizon), miR-130 family LNA inhibitors (Exiqon), and Silencer select siRNA for *Ddx6* and *Kdr* (Thermo Fisher Scientific) were used at 10 nM. Transfections were performed for 16 h followed by the removal of the transfection media. After a further 24 h, cells were passaged and used within 24–48 h. For STAT3 inhibition experiments, STAT3 Inhibitor VI, S3I-201 (Santa Cruz Biotechnology) was added to the media at 30 μM final concentration.

### Cell migration assay

ECFCs were plated on collagen-coated 24-well plates and cultured until they reached confluency. A “scratch” was then created in the monolayer with a p200 pipette tip and images of the scratch were taken with a phase contrast microscope (Nikon). Plates were placed in the hypoxia chamber and cells were allowed to migrate for 12–24 h when a second set of images was taken. The migration area was calculated as the difference in the scratch area between the initial and final time points. The video was created by concatenating images taken every 20 min using the EVOS FL2 imaging system (37°C, 80% humidity, 5% CO_2_, 1% O_2_).

### Tube-formation assay

Matrigel and media were equilibrated to low oxygen by placing them in hypoxia chamber with loose lids for 1 h prior to setting up the assay. The ECFC cell suspension (8 × 10^4^ cells per Matrigel blob) was mixed 1:1 with growth factor-reduced Matrigel (BD Biosciences), 50 μL aliquots were spotted per well of a 24-well plate. After polymerization, spots were covered with culture medium and the tube formation was assessed after 24–72 h. Tubes were then stained with Calcein AM (Thermo Fisher Scientific) and wells were imaged using a laser confocal microscope (Nikon). Total tube area was quantified using ImageJ software.

### Immunocytochemistry

Cells were fixed in 4% paraformaldehyde (PFA), blocked with 5% goat serum in PBS-T (0.1% Tween 20 in PBS) for 1 h, and incubated overnight at 4°C with primary antibodies. Antibodies used were STAT3 and phospho-STAT3 (Abcam) and VEGFR2 (Cell Signaling). To assess cell proliferation, we used staining with Ki67 antibody (BD Bioscience). After washing, cells were incubated with respective Alexa 488 secondary antibodies (Thermo Fisher Scientific) for 1 h at room temperature, mounted in Vectashield antifade medium with DAPI (Vector Labs), and imaged with a confocal microscope (Nikon).

### Clonogenic assay

ECFCs were seeded onto 6-well plates at a density of 200 cells per well and allowed to grow inside a hypoxia chamber until colonies were established (7–10 days). Colonies were then washed in PBS and stained with a solution containing glutaraldehyde 6.0% (vol/vol) and crystal violet 0.5% (wt/vol) in distilled water for 30 min at room temperature. Plates were washed by immersion in distilled water and left to dry in normal air at room temperature.

### Protein extraction and western blotting

Protein extractions were performed inside a hypoxia chamber. Cells were lysed in 1× radioimmunoprecipitation assay (RIPA) buffer containing protease and phosphatase inhibitors with EDTA (Thermo Fisher Scientific). 30 μg of protein was loaded onto SDS polyacrylamide gels. After electrophoresis, proteins were transferred to a polyvinylidene fluoride (PVDF) membrane and membranes were blocked for 1 h in clear milk (Thermo Fisher Scientific). Membranes were incubated with primary antibodies overnight at 4°C. Antibodies used were HIF1α (R&D Systems), STAT3 and phosphorylated-STAT3 (Abcam), DDX6 (Abcam), and VEGFR2 (Cell Signaling). After washing with TBST, respective horseradish peroxidase (HRP)-conjugated secondary antibodies (Bio-Rad) were applied. Blots were developed using chemiluminescent HRP substrate (Bio-Rad). For the loading control, PVDF membranes were reprobed with a monoclonal antibody to β-actin (Cell Signaling).

### Generation of miR-130 3′ UTR reporter ECFCs

Lentiviral particles were generated by transfection of HEK293T cells in a T75 tissue culture flask with 2 μg MISSION miR-130 3′ UTR Lenti GoClone vector, 1.5 μg psPAX2 packaging plasmid (Addgene #12260), and 0.5 μg pMD2.G envelope plasmid (Addgene #12259) using 15 μL of Lipofectamine 2000 (Invitrogen). Media were replaced 24 h after the transfection and virus-containing media were collected 48 and 96 h after that, filtered through a 0.45 μm filter, and stored at −80°C. Lentivirus titer was determined with the qPCR Lentivirus Titration (Titer) Kit (abm) following the manufacturer’s instructions. ECFCs were plated in 6-well plates (50,000 cells per well) and lentivirus-containing media were added to the cells the following day in the presence of 8 μg/mL polybrene (abm) at MOI equals 4. The following day, media were replaced with fresh media. For selection, cells were expanded in EGM2 containing 0.25 μg/mL puromycin for 14 days. For measurements in hypoxia, miR-130 3′ UTR reporter ECFCs and control ECFCs were plated in three white 96-well plates (4,000 cells per well, 4 replicates per cell type). Luminescence was measured using the MISSION LightSwitch Luciferase Assay Reagent (Merck, UK) following the manufacturer’s instructions. Cells were kept in PBS during the assay to reduce background signal. Simultaneously, cells were cultured in clear 96-well plates under the same conditions, stained with DAPI after 48 h, and counted to allow for normalization of luminescence signal to total cell number.

### Flow cytometry

Cells were trypsinized, collected, and incubated with antibodies for VEGFR2, CD31, CD34, CD105, and CD29 (eBioscience). Controls for gating strategy included isotype controls and unstained samples. After washing in PBS, cells were resuspended in 1 mL of fluorescence-activated cell sorting (FACS) staining buffer for analysis using an Attune acoustic cytometer (Thermo Fisher Scientific). At least 20,000 events were acquired for each sample, and FlowJo software was used for data analysis.

### *Ex vivo* sprouting choroidal explant model

C57BL/6 mouse pups at P8 were sacrificed and eyes were enucleated and placed in ice cold EGM-2 media. The choroid was surgically removed and sectioned using a 1 mm trephine in approximately 6 explants per eyecup. 1 × 10^4^ ECFCs/blob were resuspended in EGM-2 media supplemented with 10% FBS and mixed 2:3 with growth-factor-reduced Matrigel (Corning). Each choroid explant was suspended in Matrigel and 50 μL spotted per well of a 24-well plate and covered with media. Images were taken 5 days later. Sprouting distance was determined by measuring the distance from the periphery of the explant to the edge of the sprouting front at the four cardinal points, and values were averaged.

### *In vivo* Matrigel subcutaneous implant assay

All animal experiments were performed in conformity to UK Home Office Regulations (PPL2729) and with authorization from Queen’s University Belfast Animal Welfare and Ethical Review Body (AWERB). 8-week-old male athymic nude mice (Harlan Laboratories) were used. 6 × 10^5^ ECFCs transfected with miR-130a mimics or control mimics were resuspended in 25 μL of phenol red-free DMEM and 25 μL of growth-factor-reduced Matrigel (Corning) and injected subcutaneously under isoflurane anesthesia. After 10 days, mice were euthanized using sodium pentobarbital. Matrigel implants were removed and fixed in 4% PFA overnight. Fixed Matrigel implants were then embedded in paraffin and 10 μm sections were stained with hematoxylin & eosin (H&E) and anti-human CD31 antibody (Abcam). Sections through the middle of each blob were assessed and the number of perfused blood vessels and the area of the CD31 staining per section were quantified.

### Murine model of retinal ischemia

OIR was induced in C57BL/6 wild-type mice as previously described.^13^ Briefly, on postnatal day 7 (P7), mouse pups with their nursing dams were exposed to 75% O_2_ (Pro-Ox 110 Chamber Controller, Biospherix, Redfield, NY, USA) for 5 days. At P12, mice were returned to room air and they received an intravitreal injection of 1 × 10^4^ ECFCs transfected with miR-130a mimics or control mimics into their right eyes in a 1 μL volume. As a control, 1 μL of phenol red-free DMEM containing no growth factors or serum was injected into the left eye of each pup. Injections were performed under anesthesia induced by intraperitoneal injection of ketamine and xylazine. Cells were administered intravitreally using a 10 μL Flexifil syringe fitted with a 33G needle (WPI, Sarasota, FL). Pups were euthanized at P14 using sodium pentobarbital, and the whole eyes were fixed in 4% PFA for 1 h. Retinal flat mounts were stained with isolectin B4 (Sigma) and streptavidin-Alexa 488 (Thermo Fisher Scientific). Stained retinas were visualized and area quantification was performed using ImageJ software.

### Seahorse glycolysis assay

ECFCs were transfected with miR130a or control mimic on day 1. On day 2, ECFCs were passed and on day 3 placed inside a hypoxia chamber at 1% O_2_ concentration. On day 4, cells were seeded into Seahorse XFe96 cell culture plates (Agilent, Santa Clare, CA, USA) at a density of 1 × 10^4^ cells per well with 12 replicates per condition. Seeding was performed inside the hypoxia chamber. On day 5, Seahorse XF Glycolysis Stress Test Assay (Agilent) was performed under hypoxic conditions with the Seahorse XFe96 Analyzer (Agilent) placed inside a hypoxia chamber at 3% O_2_ concentration following manufacturer’s instructions. Injection solutions were used at the following concentrations: glucose: 10 mM, oligomycin 1 μM and 2D glucose 50 mM. Seahorse XF base medium (Agilent, part number: 103335-100) supplemented with 2 mM L-glutamin (Agilent, part number 103579-100) was used during the assay. After the assay, cells were fixed with 4% PFA for 15 min and washed with PBS. Cells were stained with DAPI and imaged on EVOS Cell Imaging System. Extracellular acidification rate measurements were normalized per 5000 cells. The Seahorse XF Glycolysis Stress Test Report Generator was used to calculate Glycolysis and Glycolytic Capacity following the manufacturer’s instructions.

### Statistical analysis

Statistical significance for comparison between two groups was evaluated using Prism software and unpaired two tailed t test analysis. For multiple comparisons, we used one-way analysis of variance (ANOVA) with posthoc testing. For the OIR study, data were evaluated using paired two-tailed t test analysis. Data visualization was performed using the R package ggplot2 and represented using dot plots, bar plots, or boxplots, as appropriate.

### Data availability

RNA-seq data have been deposited in GEO: GSE142123.

## Supplemental information

Document S1. Figures S1–S11 and Tables S1 and S2

Document S2. Article plus supplemental information
