# Supplementary material for: miR-130a activates the VEGFR2/STAT3/HIF1α axis to potentiate the vasoregenerative capacity of endothelial colony-forming cells in hypoxia
Source: Mol Ther Nucleic Acids. 2021 Jan 20;23:968–81. doi: 10.1016/j.omtn.2021.01.015 (PMC7869000; doi:10.1016/j.omtn.2021.01.015)
Supplement: Document S1. Figures S1–S11 and Tables S1 and S2 [file mmc1.pdf]

OMTN, Volume *23*

Supplemental Information

miR-130a activates the VEGFR2/STAT3/HIF1α axis to potentiate the vasoregenerative capacity of endothelial colony-forming cells in hypoxia

Jasenka Guduric-Fuchs, Edoardo Pedrini, Judith Lechner, Sarah E.J. Chambers, Christina L. O'Neill, Joana Mendes Lopes de Melo, Varun Pathak, Rachel H. Church, Stuart McKeown, James Bojdo, Kiran J. Mcloughlin, Alan W. Stitt, and Reinhold J. Medina

TITLE: MiR-130a activates the VEGFR2/STAT3/HIF1α axis to potentiate the vasoregenerative capacity of endothelial colony forming cells in hypoxia

SUPPLEMENTARY FIGURES

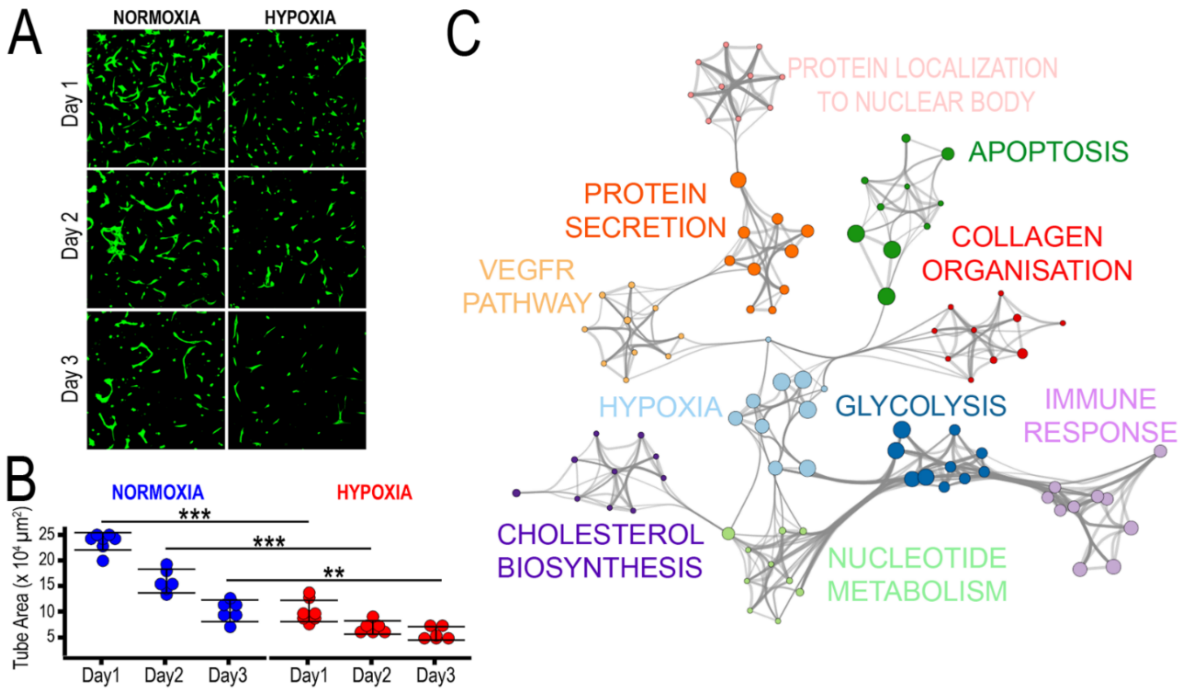

Figure S1. Hypoxia diminishes ECFC tube forming capacity. (A) Representative images of ECFCs cultured for 72 hours under hypoxia or normoxia using the Matrigel 3D tube formation assay. ECFCs were labeled with calcein-green for visualization by an epifluorescent microscope. **(B)** Quantification and statistical analysis comparing tube areas in normoxia vs. hypoxia for each time point. **$p<0.01$, ***$p<0.001$ **(C)** Gene Ontology analysis network for transcripts that were differentially expressed when comparing ECFCs cultured for 24 hours under hypoxia vs. normoxia.

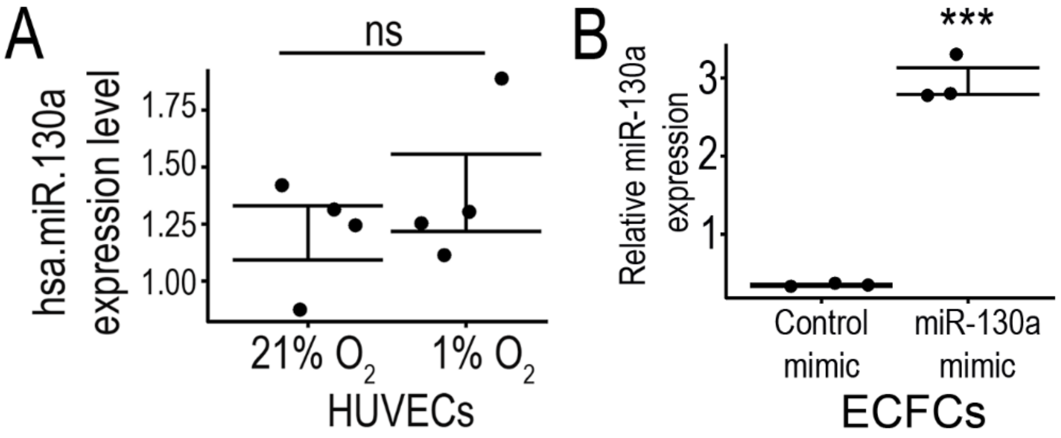

Figure S2. Hypoxia does not change miR-130a expression in HUVECs. (A) Analysis of miR-130a expression based on GEO dataset GSE17944, which investigated the response of human umbilical vein endothelial cells (HUVECs) to 1% O_2. ns: not significant. **(B)** Taqman-PCR to evaluate miR-130a expression in ECFCs after treatment with miR-130a mimics or control mimics. ***p<0.001

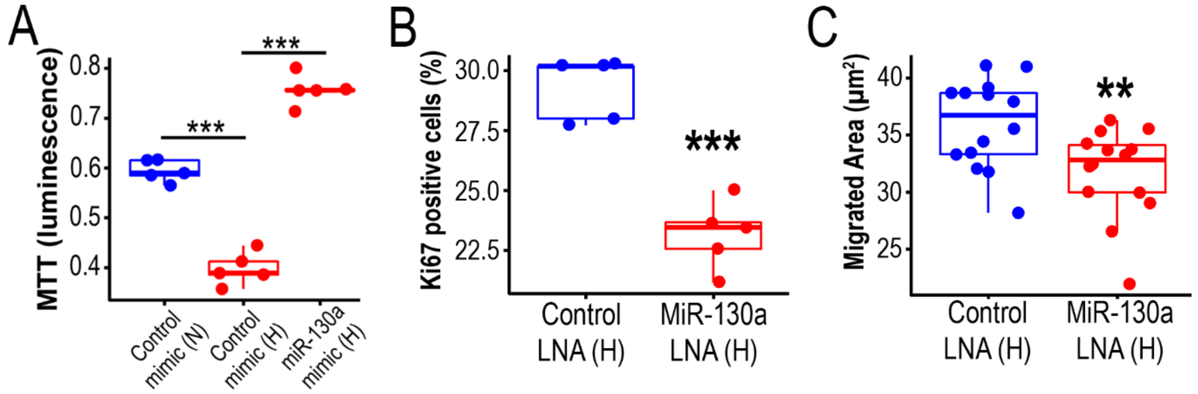

Figure S3. Blocking miR-130a is detrimental to ECFC function. (A) Survival assay by serum starvation in 0.1% FBS for 24 hours in ECFCs that were treated with control mimics or miR-130a mimics and exposed to hypoxia. MTT reagent was added for the last 4 hours to identify viable cells by measuring luminescence. **(B)** ECFCs were treated with miR-130-LNAs or control-LNAs, before exposing them to hypoxia. Ki67 expression as percentage of positive cells was quantified after 24 hours exposure to hypoxia. **(C)** Similarly, cell migration was quantified in a scratch wound assay in ECFCs treated with miR-130-LNAs or control-LNAs under hypoxia. Normoxia (N), Hypoxia (H) **p<0.01, ***p<0.001, ns: not significant.

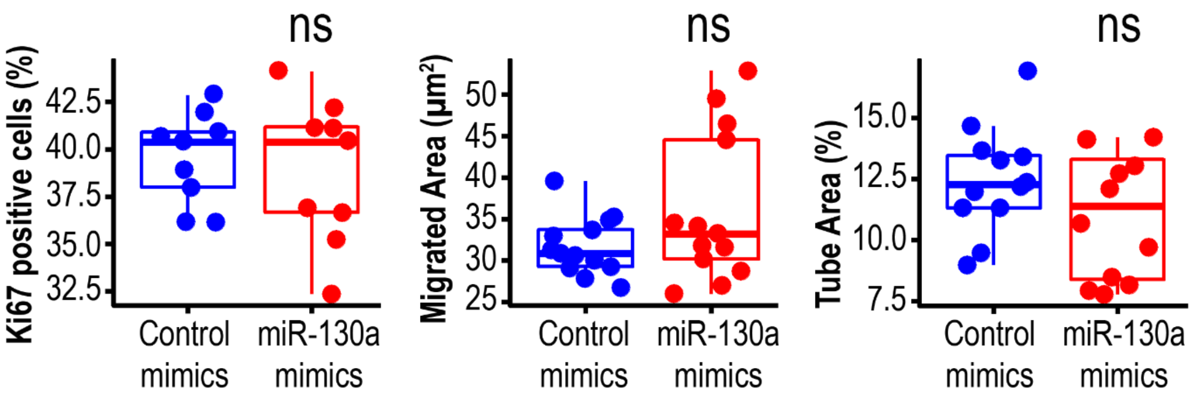

Figure S4. MiR-130a overexpression does not enhance ECFC functionality under normoxia. ECFCs treated with miR-130a mimics or control mimics under normoxia were assessed for Ki67 expression, cell migration, and tube formation. ns: not significant.

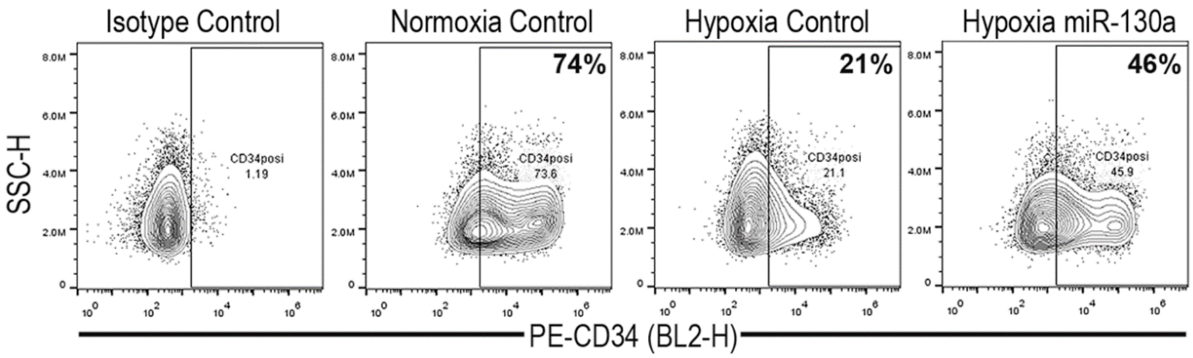

Figure S5. MiR-130a mimics restore CD34 expression in hypoxic ECFCs. Representative contour plots for flow cytometry data evaluating CD34 expression in ECFCs treated with miR-130a mimics under hypoxia. Positive gate was established by the isotype control stained sample. Percentage of positive cells is shown on the top right of the gate.

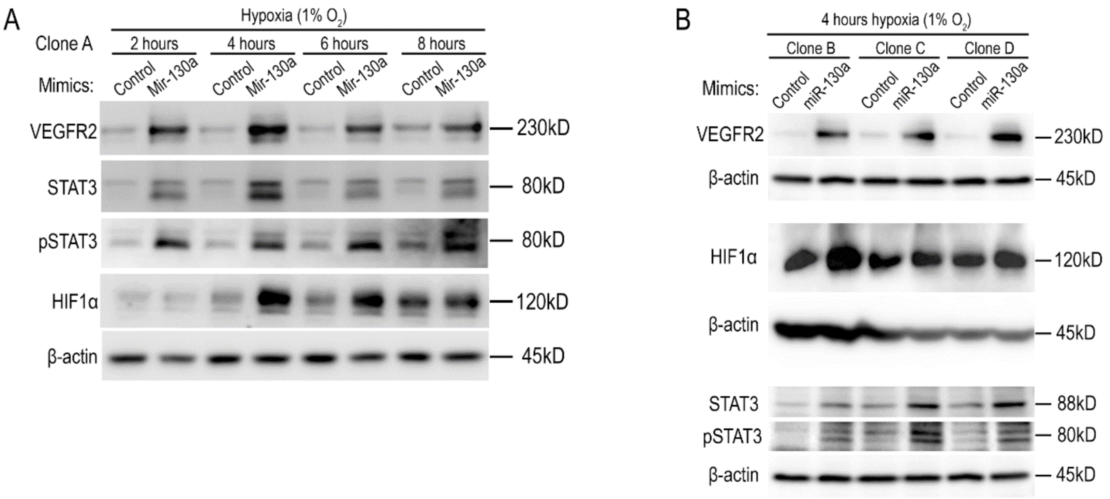

Figure S6. MiR-130a mimics increase VEGFR2, HIF1α, STAT3, and pSTAT3 in hypoxic ECFCs. (A) Western blot analysis of VEGFR2, STAT3, phosphorylated STAT3, and HIF1α in the protein extract of miR-130a and control mimics-transfected ECFCs after 2, 4, 6 and 8 hours exposure to hypoxia. **(B)** Extended western blot data of replicates used for statistical comparison in Figure 4D.

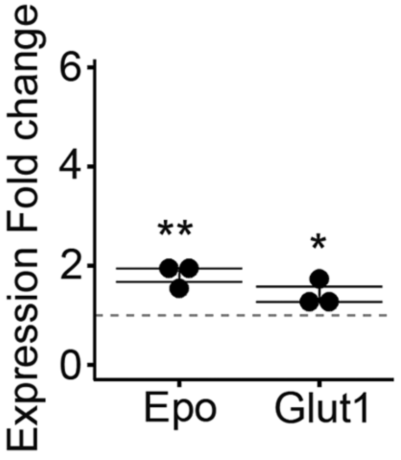

Figure S7. MiR-130a mimics increase expression of Epo and Glut1 genes. RT-qPCR to assess the expression of HIF1α target genes Epo and Glut1 in ECFCs treated with miR-130a mimics in comparison to control mimics, under hypoxia conditions. *p<0.05, **p<0.01

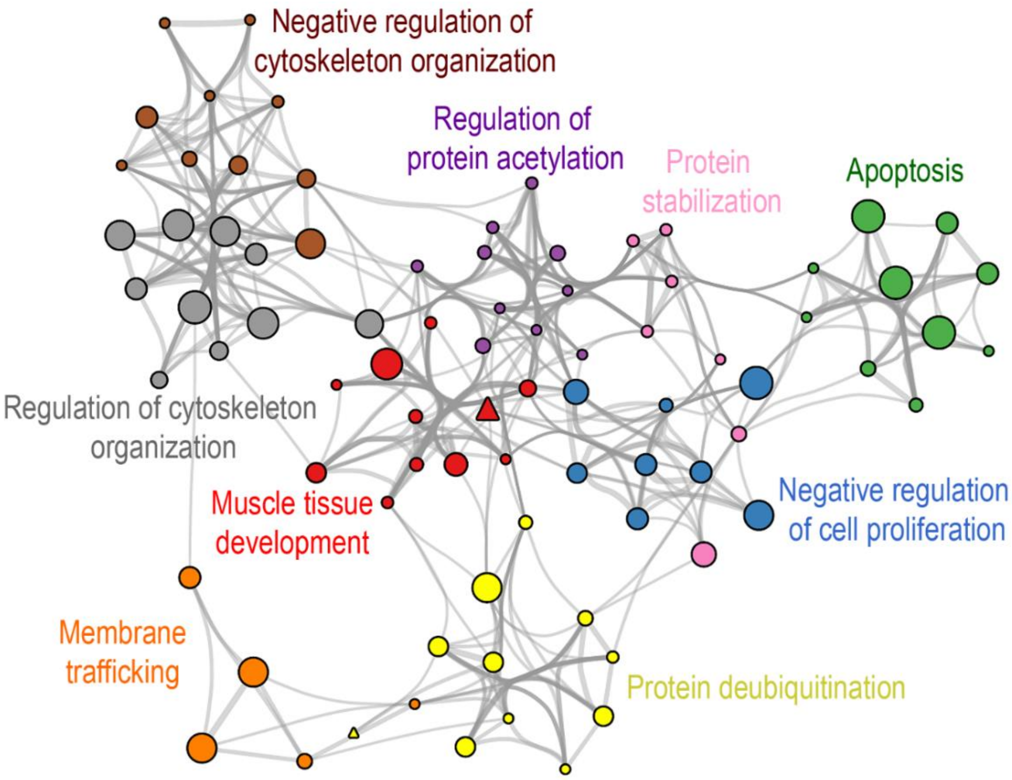

Figure S8. Cytoscape-based process enrichment analysis of miR-130a target genes. Process enrichment analysis using 185 validated miR-130a target genes. Network generated by Cytoscape to include the top clusters in different colors. Triangles highlight the nodes that include DDX6.

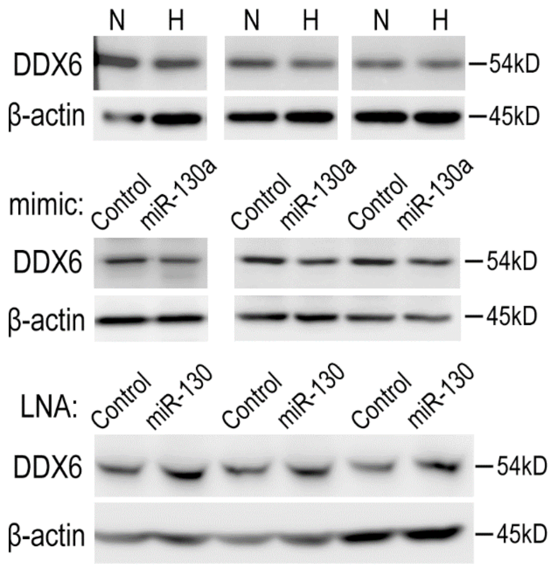

Figure S9. DDX6 expression is controlled by miR-130a. Western blot evaluation of replicates used for statistical analysis in Figure 5B.

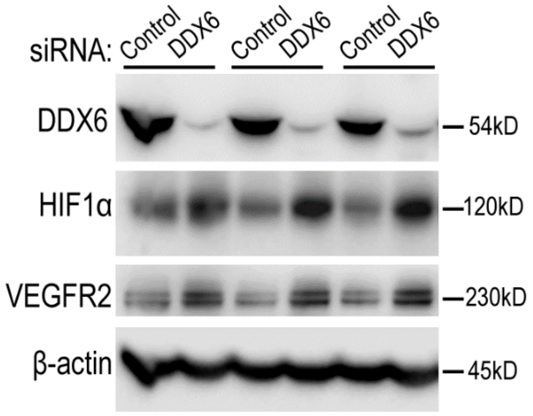

Figure S10. DDX6 silencing increased HIF1α and VEGFR2 expression. Western blot evaluation of replicates used for statistical analysis in Figure 5E.

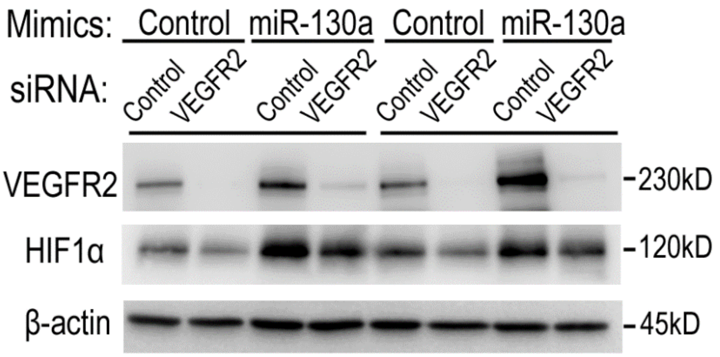

Figure S11. VEGFR2 silencing decreased HIF1α expression induced by miR-130a mimics. Western blot evaluation of replicates used for statistical analysis in Figure 5G.

Supplementary Movie 1. MiR130a mimics treatment increased ECFC migration speed under hypoxia. Dynamics of cell migration studied in ECFCs under hypoxia using the IBIDI silicone culture-inserts. ECFCs were stained with CytoPainter Cell Tracking Green and snap shots were taken every 15 minutes for 12 hours in EVOS onstage incubator.

Supplementary table S1. Output table from the Signaling Pathway Impact Analysis (SPIA) for differentially expressed genes in ECFCs exposed to hypoxia. Statistically significant top 15 enriched pathways ranked based on their significance.

Name	ID	pPERT	pG	pGFdr	Status
HIF-1 signaling pathway	4066	0.829	9.62E-07	1.20E-04	Activated
Central carbon metabolism in cancer	5230	0.108	4.65E-04	0.0290	Inhibited
Renal cell carcinoma	5211	0.024	0.0011	0.0459	Activated
AGE-RAGE signaling pathway in diabetic complications	4933	0.232	0.0031	0.0747	Inhibited
Relaxin signaling pathway	4926	0.131	0.0041	0.0747	Inhibited
ECM-receptor interaction	4512	0.522	0.0042	0.0747	Inhibited
Fluid shear stress and atherosclerosis	5418	0.114	0.0046	0.0747	Inhibited
Cholesterol metabolism	4979	0.024	0.0050	0.0747	Inhibited
IL-17 signaling pathway	4657	0.06	0.0054	0.0747	Activated
PI3K-Akt signaling pathway	4151	0.606	0.0085	0.1008	Inhibited
Focal adhesion	4510	0.373	0.0089	0.1008	Activated
Dilated cardiomyopathy	5414	1	0.0098	0.1019	Inhibited
MAPK signaling pathway	4010	0.44	0.0109	0.1048	Inhibited
Rheumatoid arthritis	5323	0.168	0.0119	0.1063	Activated
Parathyroid hormone synthesis, secretion and action	4928	0.024	0.0162	0.1346	Inhibited

Supplementary table S2. Output table from the Ingenuity Pathway Analysis (IPA) for differentially expressed genes in ECFCs exposed to hypoxia. Statistically significant enriched pathways.

Ingenuity Canonical Pathways	-log(p-value)	Ratio	z-score
HIF1α Signaling	4.84E+00	3.90E-02	1.414
ILK Signaling	1.73E+00	2.11E-02	2
Sirtuin Signaling Pathway	1.70E+00	1.72E-02	-1.342
IL-8 Signaling	1.65E+00	2.00E-02	1
Hepatic Fibrosis Signaling Pathway	1.33E+00	1.36E-02	2.236
